# Nurse anaesthetists’ experiences of student nurse anaesthetist learning during clinical practice: a qualitative interview study

**DOI:** 10.1186/s12912-024-01818-y

**Published:** 2024-02-28

**Authors:** Jakob Hedlund, Karin Blomberg, Hans Hjelmqvist, Maria Jaensson

**Affiliations:** 1 Department of Anaesthesia and Intensive Care, Örebro University Hospital, Region Örebro County, Örebro, Sweden; 2 Faculty of Medicine and Health, School of Health Sciences, Örebro University, Örebro, Sweden; 3 Faculty of Medicine and Health, School of Medical Sciences, Örebro University, Örebro, Sweden

**Keywords:** Student nurse anaesthetist, Clinical practice, Interprofessional learning

## Abstract

**Background:**

The professional role of a nurse anaesthetist involves taking a pedagogical approach towards students, including supervision during clinical practice. Although supervisors are facilitators of student learning, they are offered little training in adult learning principles. The aim of this study was to describe supervisors’ experience of student nurse anaesthetist learning during clinical practice in the operating room.

**Method:**

In this qualitative interview study, 12 semi-structured individual interviews were carried out with clinical supervising nurse anaesthetists. The data were analysed inductively using thematic analysis.

**Results:**

The results are illustrated with one theme and five sub-themes. The clinical learning situation of student nurse anaesthetists is described as a reflection of different cultures coming together. The operating room environment is a new context to students, and students enter with different clinical background and experiences. There is tension in facilitating student learning due to demands for productivity; supervisors suggest the use of separate operating rooms with a special focus on learning in the future.

**Conclusion:**

Clinical practice facilitates student learning and is a parallel process to routine care. Thus, it requires the cultures of higher education and healthcare organizations to co-exist. This is illustrated with the theme “Contributing to students’ future professional roles by bridging the hospital and university cultures”. In the operating room, student learning is challenged by a new context and time pressure as shown by subthemes. To overcome challenges and support student learning in the operating room from a supervisors’ perspective, interprofessional student teams are suggested as a future approach and need to be further investigated.

**Supplementary Information:**

The online version contains supplementary material available at 10.1186/s12912-024-01818-y.

## Background

Pedagogical competence is integrated into the professional role of nurse anaesthetists (NAs). Among other things, such competence includes providing individualized information and education to patients and family members [1]. NAs’ pedagogical skills also include supervising student nurse anaesthetists (SNAs) during clinical practice [1].

Student supervision is a process that occurs in parallel with the provision of safe care to patients [2]. In the operating room (OR), supervision takes place in an advanced setting [3] with an increasing number of surgical procedures [4]. NAs’ supervisory role facilitates student learning [5]. Thus, supervising NAs are expected to act as role models, guiding and supporting students in their learning process [6]. Supervisors should support students in learning both technical and non-technical skills [7] and should provide both direct supervision (e.g. when the supervisor is present in the learning situation) and indirect supervision (e.g. when the student is allowed to work with more responsibility and less supervised observation) [8]. The supervisory role includes performing formative and summative assessments of student progression [9]. Finally, it is expected that supervisors will discuss and reflect with students in order to support the latter’s learning process [10].

Although NAs are expected to supervise students, they are provided with limited training in adult learning principles and education theory [5], and having clinical knowledge and skills does not automatically grant an NA excellence in supervision [2]. In general, supervision during clinical practice is integrated into the responsibilities of NAs and takes place in the OR together with daily work. Previous research has highlighted the use of simulation-based training to support student learning in the OR context. It has also been reported that challenges in relation to supervision and student learning in the OR include time pressure and disruptive behaviours from team members [11]. Therefore, there is value in describing student learning during clinical practice in the OR from a supervisor’s perspective.

## Method

### Aim

The aim of this research was to describe supervising NAs’ experience of SNA learning during clinical practice in the OR.

### Design

This work is a qualitative interview study. Data was collected through semi-structured interviews and data were analysed inductively using thematic analysis based on Braun & Clarke (2006). This approach was deemed appropriate to receive supervisors experiences of SNAs clinical learning situation [12].

### Setting and participants

NAs were recruited from hospitals in six counties in Sweden, with the intention of obtaining a variety of participants from hospitals in both urban and rural settings. The inclusion criteria were NAs with experience in supervising students during clinical practice. Therefore, a purposive sampling approach was performed [12]. The manager of each clinical site transmitted an invitation e-mail with two reminders to the clinic’s NAs. A weblink was attached to the e-mail, directing the NAs to a webpage created for this study. The webpage presented the aim of this study, gave the researchers’ contact information and provided NAs with a form to fill in their own contact information if they were interested in participating in the study. The NAs who agreed to be participants were then contacted by a researcher using the telephone and were given oral information about the study. A letter with detailed written information and a consent form were then sent to the participant, along with a return envelope. In total, we received 13 responses expressing interest in the study, with one dropout.

### Data collection

A semi-structured interview guide (Supplementary Material 1) was developed for this study based on a previous review [11] of learning during clinical practice for students in nurse anaesthesia programmes. During the interviews, probing questions were asked for more detailed and nuanced narratives. Demographic data was also collected during the interviews. Interviews were performed between March 2022 and June 2022, with one delayed interview due to personal reasons. One pilot interview was performed to test the questions; no revision was made to the interview guide, and the pilot interview was included in the analysis. Individual interviews (*n* = 12) were conducted, and the participants decided where they wanted to be interviewed – that is, whether face to face at, for example, their workplace (*n* = 5), or via telephone (*n* = 7). The interviews were conducted by different researchers: JH (*n* = 6), KB (*n* = 4) and MJ (*n* = 2). To ensure accuracy, all interviews were audio-recorded (Philips VoiceTracer DVT 8110) and transcribed verbatim by a professional transcriber. The interviews lasted between 22 and 76 min, with a mean time of 52 min.

### Data analysis

The data were analysed inductively via thematic analysis [13]. The transcripts were first read and reread several times to allow the authors to become familiarized with the data. Initial coding of the transcripts was performed by JH in Nvivo (Version 14). An in-vivo coding strategy was applied to make it possible to identify all variations in the data in relation to the aim. The first interview to be coded was discussed between all the authors in order to validate the coding process. Three additional coded interviews (25%) were later discussed between the authors during the coding process.

The list with the initial codes was exported from Nvivo to Microsoft Excel. The codes were then grouped together based on shared meaning to create potential sub-themes and themes; these groupings were reviewed and discussed between all the authors until consensus was reached. To illustrate the content of the sub-themes and theme and to increase trustworthiness, we provide quotations in the reporting of our findings. An example of the analysis process is shown in Table 1.

**Table 1:** Examples of the analysis process

**Dataset (quotations from interviewed NAs)**	**Initial code**	**Code**	**Sub-theme**	**Theme**
‘There are new facilities, new practical elements, everything is new, the bed the patient is lying in is new, the anaesthesia equipment, all the boxes, most things are new. So it’s something that is really difficult, sure, but they catch on fairly quickly.’ (NA4)	Everything is new	Environment	Supervising in the operating room – a new context for students	Contributing to students’future professional roles by bridging the hospital and university cultures
‘The student feels comfortable and safe, I think, that’s important. Because their backgrounds differ greatly, what they bring to the table…’ (NA9)	Students’ backgrounds differ greatly	Different needs	Adapting supervision based on students’ conditions	
‘In our activities, we are always hard pressed for time, you’d like to have the time to be able to sit down and plan things.’ (NA11)	In our activities, we are always hard pressed for time	Time	Supervising despite the mismatch between ambition and reality	
‘We do have interim assessments where one can review things so, yes, there one has an opportunity to see whether they’ve missed anything or if there’s something they need to work on more or need to take a look at. So, I feel, I find that we have had space to identify whether one has fallen behind or achieved the goals.’ (NA1)	Interim assessment offers an opportunity to see if there is anything that one needs to work on	Assessment	Guiding students through assessment	
So, I think that the peer learning that we have now done a few times in … the latter part of the workplace training has been extremely successful. The students have been very pleased, and found it to be really fun.’ (NA10)	Peer learning towards the end was successful	Peer learning	Using collaborative learning to enhance student learning	

### Ethical considerations

Ethical approval was received from the Swedish Ethical Review Authority Board (Dnr 2021-06027-01; Dnr 2022-02095-02). Participation in the study was voluntary. The participants received oral and written information before giving their written consent. The interviews were conducted by different researchers to avoid any former relationship or dependency between the interviewer and the participant. Also, in order to make the participants feel comfortable during the interview, the participants were given the choice of where the interview would take place. Interviews and transcripts were stored in a secure place that was only accessible to the researchers.

## Results

**Fig. 1:**
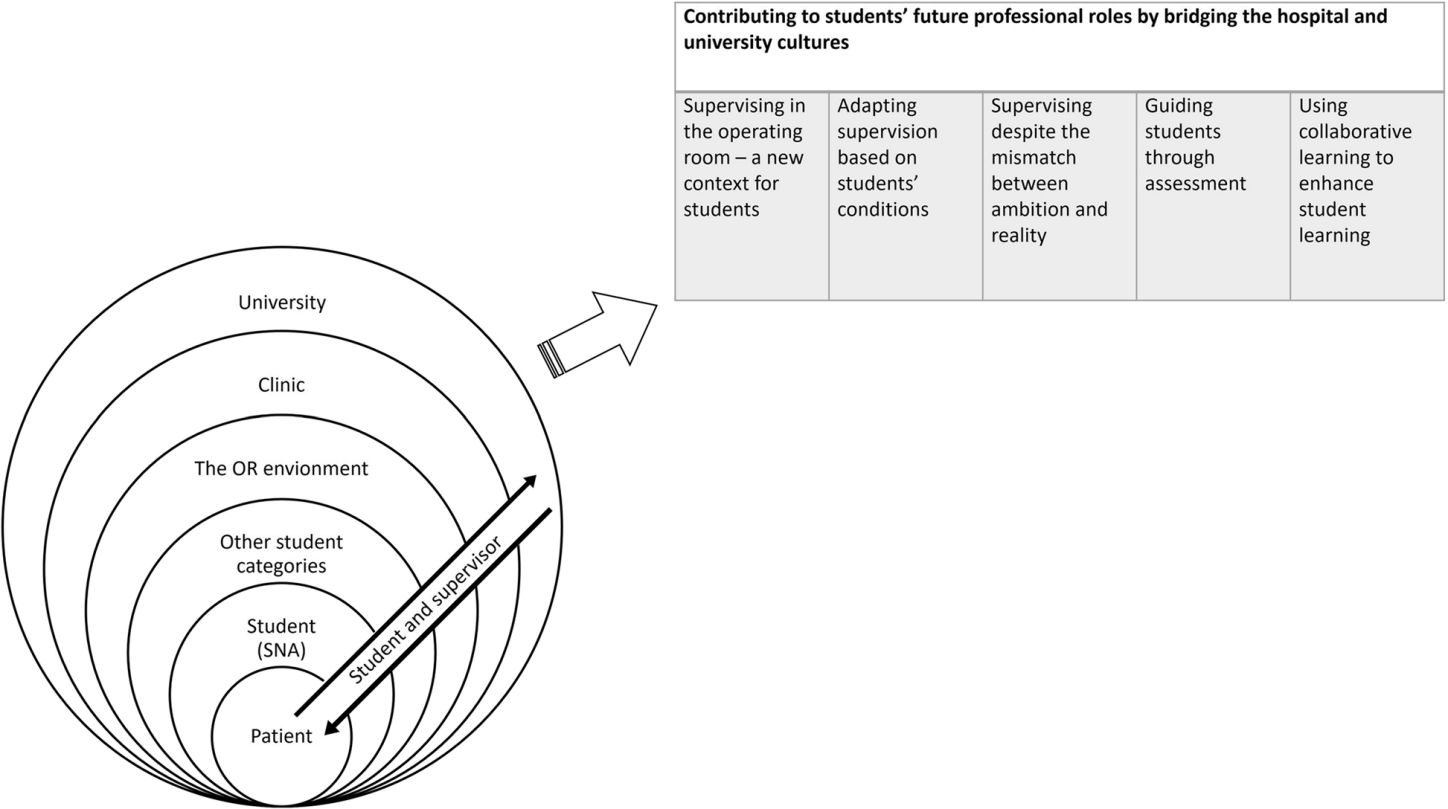
Illustration of the results with the theme and sub-themes

The final sample (*n* = 12) consisted of four men and eight women. The age of the participants varied between 37 and 63 years, with a mean age of 48.8 years. Their working experience as an NA varied between 4 and 34 years, with a mean of 14.3 years. The findings are presented in the form of one theme and five sub-themes. It was clear in the results how SNAs and supervisors handle external factors in different contexts during the learning process with a patient focused approach (Fig. 1).

### Contributing to students’ future professional roles by bridging the hospital and university cultures

In the interviews, the supervisors described how student learning interacts with external factors. The identified theme and sub-themes demonstrate how student learning is integrated with these external factors.

#### Supervising in the operating room – a new context for students

When students begin their first period of clinical practice, it can be challenging to face and learn in a new context within the healthcare environment. The supervisors described how the students need to learn the workflow and how to act in the OR. As a learning environment, the OR offered the students many new impressions and could be challenging before the students became familiar with it (e.g. the students needed to learn the rules for clothing, to ensure remaining aseptic and sterile, which implied the need for certain behaviours). The OR was also described as containing a wide range of technical equipment and many sounds. Moreover, the supervisors described the physical space in the OR as being limited and sometimes narrow. They also mentioned that, before the start of a surgery, the patients might need to be positioned in certain ways.

It is a special environment in which it takes time to get yourself oriented, so it is very clearly a challenge. And you take it a bit personally when you may have forgotten to tell them that ‘This is what applies here’. Yes, and if I haven’t told them, then the student can’t possibly know it, and you take it a bit personally, perhaps in the sense that ‘Oh heck, now the student has gotten a reprimand, which is a shame’. (NA 1)

The work around the patient is performed by OR teams. The supervisors reflected on how the OR could be experienced as a big workplace that included different professions. Therefore, it could be difficult for students to know everyone and to understand their respective responsibilities. The supervisors also talked about how the students worked simultaneously with other team members (e.g. OR nurses) in the OR and reflected on how this can make the students feel observed during supervision. Moreover, the supervisors described how the teams could be dynamic, changing with short notice.

Then I had presented … uh, so the whole team now knows that we had a student, and it was a very special patient. And then, when we were to take them in, suddenly the entire surgical team was switched out, and I didn’t know … (NA 3)

Working in teams requires communication skills. Some supervisors described the use of difficult jargon in the OR, and mentioned that the students were sometimes subjected to negative comments from other team members.

In purely concrete terms, comments such as ‘It’s taking too long’ may be made or, more subtly, in the form of a bit of moaning and groaning – and, yes, this is mainly in situations in the OR when one is anaesthetizing or wake the patient. (NA 1)

#### Adapting supervision based on students’ conditions

The supervisors reflected on how they adapt their supervision to facilitate the students’ learning. Since the students’ previous working experience varied, they entered with different prerequisites. The supervisors described encountering some students during their supervision who had never been in the OR before, while other students had experience in an acute care setting or had even worked as an undergraduate nurse in the OR. Therefore, the supervisors applied a dynamic supervision style and adjusted their supervision to meet the needs of the student. Some of the students wanted to start observing, some wanted to do a little by themselves and some wanted to do everything at once.

A person who has been in an ambulance is very familiar with hooking up and injecting patients, they don’t need to focus on that. But someone who comes from geriatric care may not have done so much of that, and they may need to take a day and keep on with that. (NA 11)

The supervisors viewed themselves as taking on a guiding role to support the students in their learning process. This role included communicating the basics of the profession and being available for discussing questions with the students.

The supervisors wanted the students to take their own initiative; the supervising NAs described how they balanced between pushing and holding back the SNAs during the supervisory role. The supervisors tried to introduce their students to the work step by step. In the beginning, the supervisors demonstrated procedures to the student, describing their use of a ‘see one, do one approach’. Later, to stimulate the students’ learning, the supervisors asked questions, encouraging the students to think aloud and to motivate their actions. The supervisors described how this approach challenged the students in their transition towards independence. For example, this approach could involve asking the student to suggest reasonable doses of different drugs, or encouraging students to go home, study, and then give a short report on their learning the next day. The supervisors encouraged the students to take the lead during patient care, such as by encouraging the students to communicate and collaborate with the anaesthesiologist, resulting in the supervisor taking on a more peripheral role.

I believe that many still appreciate it, in that one first asks, ‘how do we administer this?’ and ‘why are we administering it?’ and ‘how much?’. And ‘roughly how does it work?’. And, so, you get to think about it, and then you can discuss it further. (NA 2)

Patient safety was always a priority when supervising students. The supervisors described maintaining a balance between providing the students with learning opportunities and not risking patient safety. In this process, the supervisors explained, the planning could change quickly, and they needed to step in and take over when a situation changed rapidly – such as during acute situations or if the patient was severely ill. The NAs also described how they tried to pass on a patient safety culture to students during OR work, including never being embarrassed to ask for help in time.

#### Supervising despite the mismatch between ambition and reality

The supervisors described time as a critical factor during supervision that could make student learning challenging in the OR. The NAs described the OR as a hectic environment with a high tempo and quick changes. Supervision was described as being guided by time framing and as being positioned in relation to the schedule, with its planned surgeries for the day. For example, due to the requirement for productivity, the supervisors experienced a lack of time for planning, reflection, feedback and allowing students working at a suitable pace while supervising. However, the NAs emphasised that the supervision of students must be allowed to take time. Because of the quick tempo, the supervisors used their coffee breaks and lunch break as a forum for planning (e.g. to plan for the next case and for the upcoming day) and formative assessment.

That’s where, as a supervisor, you try to stay ahead of things and plan and look around, try to get out of the OR and plan things on your lunch and coffee breaks, because it’s so important get things right in the OR …

Then you always try to arrange things so that we can go on a coffee break or we can get out for a while, and the coffee breaks are always when we can reflect … (NA 3)

The students were viewed as future colleagues by their supervisors, who desired them to get the most out of clinical practice. At the same time, the supervisors described how they wanted to prepare their students for the tough reality they would encounter after graduation.

I want them to be prepared, in that they can’t be too soft, because this profession is tough at the start, and there can’t be too big of a gap between the supervisory period and when they enter their working lives, because then they will be in for a shock. (NA 5)

The supervisors stated that they modified their supervision based on reality, since the OR ‘is what it is’, and the environment cannot be changed. Therefore, the supervising NAs tried to make the best out of the situation. Doing so might involve letting the students start their day earlier, in order to give them enough time to prepare for the first patient case of the day; alternatively, it might involve not including the student in the first patient case of the day and instead letting the student prepare for the second patient case without stress. Because of the stressful environment, the supervisors wanted to safeguard and stand up for their students in their learning process.

Sometimes, when you sense that … that the others may lack understanding, then I will come to the defence of my student … but, just like you want to advocate for the patient, so you also become the student’s advocate sometimes. I think that can be necessary. (NA 6)

#### Guiding students through assessment

The supervisors described the role of assessment in guiding student learning. The students had their own responsibility for their learning: it was the students’ responsibility to be familiar with the learning outcomes, to clarify individual learning needs and to become familiar with essential theory. It was perceived as a hindrance when students were not prepared regarding theory. However, the supervisors generally experienced the students as having good theoretical knowledge upon arrival.

I’ve been supervising for so many years, so I think that they are, they are very well informed … I feel that I’m very much at the bottom in terms of theory, very much so. But they really know their theory … (NA 4)

The supervisors described how they provided formative assessment to support student learning. Some supervisors wanted to have time to sit down to talk with students in private, but it was not always possible to find the time. Other supervisors tried to continuously reflect on progression together with students during the day; some sat down with students at the end of the day, while others found a gap in time between patient cases.

The supervisors emphasised the importance of identifying early on whether there was a risk that the students would not achieve the intended learning outcomes. This identification usually occurred during the half-time assessment, half-way through the clinical practice. The assessment was described as being positive; it could function as a support for further planning and guiding the students’ learning.

Regarding the summative assessment at the end of the clinical practice, the supervisors stated that they followed the intended learning outcomes that were specified in the assessment tool provided by the faculty. Some supervisors had been involved in assessments they described as frustrating; such assessments occurred when there were different opinions between the supervisors and the faculty teacher regarding whether the students had reached the intended learning goals or not. The assessment was experienced as being difficult in general; the supervisors noted that it was helpful to have a co-supervisor to support the main supervisor during the assessment, although students were not always assigned two supervisors.

#### Using collaborative learning to enhance student learning

The supervisors described a sense of competition among students to obtain access to patient cases, particularly in regard to training in airway management. There are other students categories, novice nurse anaesthetists and physicians in the OR, all of which want to practice airway management.

We have a whole lot of interns, we have resident anaesthesiologists … they are also very important, they have to get their … airways to have access to. So, it is really crowded getting access here to operations, advanced ambulance training students want to come here, they come from the helicopter and all of them want access to this airway … (NA 3)

To address the challenges in the students’ clinical learning, several supervisors expressed a desire for a more student-friendly and customized OR. Such an OR could allow students from different professions to work together. The supervisors reflected on the idea of an OR with a reduced operating schedule and fewer planned surgeries, which would allow students to work in a slower pace. The supervisors also reflected on how this type of OR could include easier patient cases (i.e. patients that were not severely ill).

I wish that we had student ORs there, where you could focus specifically on the simpler interventions, ambulatory surgery, and you would know that, in this OR, here, we will be supervising. And then there could be surgical nursing students in the OR at the same time, and then you’d get a greater … uh, then you’d get a better supervising situation. And then you’d know, then you’d have a limited number of patients, and I believe that this would offer more, in fact, it would be better for everyone. (NA 13)

Furthermore, the supervisors described the use of peer learning as a model that could enable supervision to be better appreciated by students and could give students the opportunity to act while carrying responsibility. Peer learning was described as being more suitable in the later part of clinical practice, when the students become more independent; such learning would require appropriate cases for students to handle independently. Several supervisors reflected on the advantages and disadvantages of peer learning, with some supervisors having more experience in this area than others.

## Discussion

The analysis revealed that, when the supervisors discussed students learning during clinical practice in the OR, lack of time was a recurring topic. To overcome challenges during the student learning process and create a balanced learning situation, the supervisors argued for the introduction of interprofessional learning to the OR by having students from various streams of learning work together in the same OR.

With the patient in focus, the supervisors described layers or dimensions that interacted with student learning (Fig. 1). Student learning could be challenged by the new context, competition in cases with other student categories, interactions with other professions in the OR team and the assessment of learning goals. Students’ clinical practice is a complex collaboration between higher education and healthcare organizations, who rely on each other to educate healthcare professionals [14]. Each setting (i.e. higher education and healthcare organizations) has its own thoughts, traditions and norms; taken together, these shape the OR learning context and must be adapted to mesh with those of the other organization(s) [14]. From a supervisor’s perspective, collaboration and communication between the faculty and the clinical team members are critical when planning supervision. The expectations for the supervisory role must be clarified, along with practical details such as what the students are allowed to do and how to handle events that fall outside the planned scenarios [15]. However, the clinical management sometimes failed to give the supervising NAs the prerequisites they needed to supervise (e.g. time to supervise due to demanding working situation) [16].

The analysis revealed that the supervisors found that the students entered the new context of the OR with different prerequisites. A organization’s culture is shaped by its goals, values, leadership and finances, among other things [17]. To stimulate learning in the perioperative setting, a culture of trust (e.g. a structured introduction), respect (e.g. a blame-free environment) and communication (e.g. an environment where speaking up is encouraged) support students in learning skills and competencies [18].

The analysis showed that insufficient time was a concerning factor during supervision that negatively affected planning, reflection and feedback. This finding is in line with the results of previous studies [19, 20]. The supervisors demonstrated high flexibility and a commitment to support student learning, as exemplified in their descriptions of using their own breaks for planning due to the shortage of time. This finding can be put in relation to the global issue of the shortage of nurses [21], as a shortage of time implies fewer supervisors. Also, to maintain staffing, hospital managers use alternative work arrangements (e.g. travelling nurses) for short time assignments [22]. To the best of our knowledge, there are no studies describing how alternative work arrangements affect student learning. Moreover, supervision is a common element in the everyday work of nurse anaesthetists [23], and a previous study has shown that the profession of nurse anaesthetists is the most likely to multitask and to be interrupted, which also applies when supervising [24].

To better support students and overcome challenges in supervision, the supervising NAs reflected on the possibility of having an OR with a special focus on student learning. Such an OR could include students from all OR team professions learning together, with fewer planned surgeries. The concept of students from two or more professions learning about, from and with each other is called *interprofessional education* [25]. However, interprofessional education is not only about gathering students from different professions; it also requires planning and commitment [26]. Previous studies on undergraduate students in interprofessional training wards describe this type of training as a cost-effective [27] way for students to learn their respective professions and teamwork [28] without endangering patient safety [29]. Interprofessional education can also increase students’ self-efficacy [30].

In the OR, interprofessional teamwork is vital to patient safety [31]. However, to the best of our knowledge, the literature on clinical interprofessional education with student teams in the OR is limited and focuses on interprofessional simulation-based training [32, 33]. Therefore, interprofessional education in the OR with student teams could be an area for future study.

The interaction of the cultures of healthcare and higher education and the concept of interprofessional learning can be theocratized using a boundary-crossing perspective rooted in the third generation of activity theory [34]. Boundaries can be defined as ‘sociocultural differences that give rise to discontinuities in interaction and action’ [35]. These boundaries, or differences, can provide opportunities for learning [35]. Learning situations occur when students are challenged to cross boundaries by combining different contexts to create a hybrid solution [36].

### Strengths and limitations of this study

To ensure credibility, open-ended questions were asked during the interviews, followed by probing questions. Also, all the researchers, who represented different professions with a different pre-understanding of the perioperative context, were included in the coding and analysis of data [37]. To ensure dependability, a semi-structured interview guide was used; in the Method section, a detailed description is provided on how the data were collected and analysed [37]. The interviews were recorded and transcribed by a professional transcriber. The transcripts were then matched against the audio file before the start of the analysis to ensure confirmability [37]. Examples from the analysis (Table 1) were presented to ensure transferability [37].

The limitations of this study may lie in its use of purposive selection. The managers in various anaesthesia departments helped to send out invitation e-mails and reminders. This approach gave the researchers limited control over the recruitment process, and the managers could have acted as gatekeepers [38] by selecting participants with a more positive approach.

## Conclusion

Students clinical practice is a facilitator to student learning and is a parallel process to routine care. Thus, it requires the cultures of higher education and healthcare organizations to coexist. This is illustrated with the theme “Contributing to students’ future professional roles by bridging the hospital and university cultures”. In the OR, student learning is challenged by the new context and time pressure as shown by subthemes. To overcome challenges and to support student learning from a supervisor’s perspective, interprofessional student teams are suggested as a future approach and need to be further investigated.

### Supplementary Information

**Supplementary Material 1.**

## Data Availability

Due to the confidentially of the participants of the current study, dataset and analysis generated are not publicly available. The ethical approval does not include this. The corresponding author can be contacted upon reasonable request.

## Abbreviations

NA: Nurse anaesthetist
OR: Operating Room
SNA: Student Nurse Anaesthetist

## References

[1] The National Association for Anaesthesia and Intensive Care & The Swedish Nurses’ Association. (Swedish: Riksföreningen för anestesi och intensivvård & Svensk sjuksköterskeförening). Kompetensbeskrivning. Avacerad nivå. Specialistsjuksköterska med inriktning mot anestesisjukvård. 2021. https://swenurse.se/download/18.b986b9d1768421a1b57604a/1610609299643/Kompetensbeskrivning%20Anestesisjuksk%C3%B6terska.pdfpetensbeskrivning%20Anestesisjuksköterska.pdf. Accessed 22 Nov 2023.

[2] Griffiths M, Creedy D, Carter A, Donnellan-Fernandez R (2022). Systematic review of interventions to enhance preceptors’ role in undergraduate health student clinical learning. Nurse Educ Pract.

[3] Fynes E, Martin D, Hoy L, Cousley A (2014). Anaesthetic nurse specialist role: leading and facilitation in clinical practice. J Perioper Pract.

[4] Yin L, Shui X, Zuo J, Yang Q, Jiang X, Liao L (2021). No harm found when the scope of practice of nurse anesthetists is expanded to the whole process of anesthetic care and under indirect supervision of anesthesiologists: a time series study. Int J Nurs Stud.

[5] Elisha S, Rutledge DN (2011). Clinical education experiences: perceptions of student registered nurse anesthetists. AANA J.

[6] Hilli Y, Melender HL, Salmu M, Jonsén E (2014). Being a preceptor—a Nordic qualitative study. Nurse Educ Today.

[7] Jones CPL, Fawker-Corbett J, Groom P, Morton B, Lister C, Mercer SJ (2018). Human factors in preventing complications in anaesthesia: a systematic review. Anaesthesia.

[8] Honkavuo L (2020). Nursing students’ perspective on a caring relationship in clinical supervision. Nurs Ethics.

[9] Elisha S, Bonanno L, Porche D, Mercante DE, Gerbasi F (2020). Development of a common clinical assessment tool for evaluation in nurse anesthesia education. AANA J.

[10] Sundler AJ, Blomberg K, Bisholt B, Eklund A, Windahl J, Larsson M (2019). Experiences of supervision during clinical education among specialised nursing students in Sweden: a cross-sectional study. Nurse Educ Today.

[11] Hedlund J, Blomberg K, Hjelmqvist H, Jaensson M (2023). Student nurse anesthetists’ and supervisors’ perspectives of learning in the operating room: an integrative review. J Perianesth Nurs.

[12] Patton MQ. Qualitative research & evaluation methods: integrating theory and practice. 4th ed. California: SAGE Publications Inc; 2015.

[13] Braun V, Clarke V (2006). Using thematic analysis in psychology. Qual Res Psychol.

[14] Bivall AC, Gustavsson M, Lindh FA (2021). Conditions for collaboration between higher education and healthcare providers organising clinical placements. High Educ Ski Work-based Learn.

[15] Landmark BT, Hansen GS, Bjones I, BØhler A (2003). Clinical supervision - factors defined by nurses as influential upon the development of competence and skills in supervision. J Clin Nurs.

[16] Ehrenberg AC, Häggblom M (2007). Problem-based learning in clinical nursing education: integrating theory and practice. Nurse Educ Pract.

[17] Sheehan D, Wilkinson TJ (2022). Widening how we see the impact of culture on learning, practice and identity development in clinical environments. Med Educ.

[18] Bello C, Filipovic MG, Andereggen L, Heidegger T, Urman RD, Luedi MM (2022). Building a well-balanced culture in the perioperative setting. Best Pract Res Clin Anaesthesiol.

[19] Jans J, Falk-Brynhildsen K, Salzmann-Erikson M (2021). Nurse anesthetists’ reflections and strategies when supervising master’s students. Nurse Educ Pract.

[20] Averlid G, Høglund JS (2020). The operating room as a learning arena: Nurse anaesthetist and student nurse anaesthetist perceptions. J Clin Nurs.

[21] Marć M, Bartosiewicz A, Burzyńska J, Chmiel Z, Januszewicz P (2019). A nursing shortage – a prospect of global and local policies. Int Nurs Rev.

[22] Gan I (2020). How do nurse managers describe clinical nurses’ work arrangements? A qualitative study. Nurs Open.

[23] Olin K, Göras C, Nilsson U, Unbeck M, Ehrenberg A, Pukk-Härenstam K, Ekstedt M (2022). Mapping registered nurse anaesthetists’ intraoperative work: tasks, multitasking, interruptions and their causes, and interactions: a prospective observational study. BMJ Open.

[24] Göras C, Olin K, Unbeck M, Pukk-Härenstam K, Ehrenberg A, Tessma MK, Nilsson U, Ekstedt M (2019). Tasks, multitasking and interruptions among the surgical team in an operating room: a prospective observational study. BMJ Open.

[25] World Health Organization [WHO]. Framework for action on interprofessional education & collaborative practice. 2010. https://www.who.int/publications/i/item/framework-for-action-on-interprofessional-education-collaborative-practice. Accessed 22 Nov 2023.

[26] Schot E, Tummers L, Noordegraaf M. Working on working together. A systematic review on how healthcare professionals contribute to interprofessional collaboration. J Interprof Care. 2020. 10.1080/13561820.2019.1636007. 10.1080/13561820.2019.1636007 31329469

[27] Hansen TB, Jacobsen F, Larsen K (2009). Cost effective interprofessional training: an evaluation of a training unit in Denmark. J Interprof Care.

[28] Jacobsen F, Fink AM, Marcussen V, Larsen K, BæK HT (2009). Interprofessional undergraduate clinical learning: results from a three year project in a Danish Interprofessional Training Unit. J Interprof Care.

[29] Kuner C, Doerr-Harim C, Feißt M, Klotz R, Heger P, Probst P, Strothmann H, Götsch B, Schmidt J, Mink J, Mitzkat A, Trierweiler-Hauke B, Mihaljevic AL. Clinical outcomes of patients treated on the Heidelberg interprofessional training ward vs. care on a conventional surgical ward: a retrospective cohort study. J Interprof Care. 2022. 10.1080/13561820.2021.1975667. 10.1080/13561820.2021.1975667 35297739

[30] Nørgaard B, Draborg E, Vestergaard E, Odgaard E, Jensen DC, Sørensen J (2013). Interprofessional clinical training improves self-efficacy of health care students. Med Teach.

[31] Mazzocco K, Petitti DB, Fong KT, Bonacum D, Brookey J, Graham S, Lasky RE, Sexton JB, Thomas EJ (2009). Surgical team behaviors and patient outcomes. Am J Surg.

[32] Paige JTMDF, Garbee DDP, Kozmenko VMD, Yu QP, Kozmenko LBSN, Yang TMD, Bonanno LDNP, Swartz WP. Getting a head start: high-fidelity, simulation-based operating room team training of interprofessional students. J Am Coll Surg. 2014. 10.1016/j.jamcollsurg.2013.09.006. 10.1016/j.jamcollsurg.2013.09.006 24183570

[33] Leithead J, Garbee DD, Yu Q, Rusnak VV, Kiselov VJ, Zhu L, Paige JT (2019). Examining interprofessional learning perceptions among students in a simulation-based operating room team training experience. J Interprof Care.

[34] Engeström Y, Sannino A (2021). From mediated actions to heterogenous coalitions: four generations of activity-theoretical studies of work and learning. Mind Cult Act.

[35] Akkerman SF, Bakker A (2011). Boundary crossing and boundary objects. Rev Educ Res.

[36] Engeström Y, Engeström R, Kärkkäinen M (1995). Polycontextuality and boundary crossing in expert cognition: learning and problem solving in complex work activities. Learn Instr.

[37] Korstjens I, Moser A. Series: practical guidance to qualitative research. Part 4: trustworthiness and publishing. Eur J Gen Pract. 2018. 10.1080/13814788.2017.1375092. 10.1080/13814788.2017.1375092 PMC8816392 29202616

[38] Clark T (2011). Gaining and maintaining access: exploring the mechanisms that support and challenge the relationship between gatekeepers and researchers. Qual Soc Work.

