# Supplementary material for: Nurse anaesthetists’ experiences of student nurse anaesthetist learning during clinical practice: a qualitative interview study
Source: BMC Nurs. 2024 Feb 28;23:141. doi: 10.1186/s12912-024-01818-y (PMC10902972; doi:10.1186/s12912-024-01818-y)
Supplement: Supplementary file 1 — Supplementary Material 1. [file 12912_2024_1818_MOESM1_ESM.docx]

| Can you tell about your experience in supervising student nurse anaesthetists? |
| --- |
| How do you experience the learning environment during clinical practice? |
| Can you describe hindrance when supervising? |
| Can you describe opportunities when supervising? |
| How can student learning be improved during clinical practice? |
| Describe your state of mind during supervision. |
| Can you tell about your experience of combining your professional role with your supervisory role? |

**Supplementary Material 1.** Interview guide
